# Intraoperative Hypotension After Neuraxial Anesthesia: A Systematic Review and Meta-Analysis of Randomized Controlled Trials of Vasopressors for Cesarean Birth Stratified by Maternal Risk

**DOI:** 10.7759/cureus.94900

**Published:** 2025-10-19

**Authors:** Arya Babul, Sohi Ashraf, Jyoti Desai, Leanne Free, Momina Hussain, Najib Babul

**Affiliations:** 1 Biomedical Sciences, West Career and Technical Academy, Las Vegas, USA; 2 Intensive Care, MountainView Hospital, Las Vegas, USA; 3 Department of Gynecologic Surgery and Obstetrics, Kirk Kerkorian School of Medicine at the University of Nevada, Las Vegas, Las Vegas, USA; 4 Genomics, Chinese Academy of Tropical Agriculture Sciences, Sanya, CHN; 5 Drug Development, Cinergen, LLC, Las Vegas, USA

**Keywords:** cesarean section, ephedrine, epidural anesthesia, neuraxial anesthesia, norepinephrine, obstetric complications, phenylephrine, post-operative pain management, postspinal hypotension, spinal anesthesia

## Abstract

Postspinal hypotension is a frequent complication during cesarean delivery, particularly in high-risk pregnancies. Although vasopressors are routinely used for its management, their relative efficacy and safety after the onset of hypotension remain uncertain. Following PROSPERO registration (CRD420251050321) and in accordance with the Preferred Reporting Items for Systematic Reviews and Meta-Analyses (PRISMA) 2020 guidelines, this systematic review and meta-analysis evaluated the efficacy and safety of norepinephrine, phenylephrine, and ephedrine administered for treatment, rather than prophylaxis, of postspinal hypotension during cesarean delivery. A comprehensive search of PubMed, Embase, CENTRAL, and Scopus through April 2025 was performed using a combination of MeSH and free-text terms related to cesarean section, spinal anesthesia, hypotension, and vasopressors. Randomized controlled trials (RCTs) comparing intravenous bolus or infusion administration of these agents in parturients undergoing high-risk cesarean delivery were included, while prophylactic trials were excluded. Two reviewers independently screened studies, extracted data, and assessed risk of bias using the Cochrane Risk of Bias 2.0 tool. Quantitative synthesis was performed using a random-effects model, and subgroup and sensitivity analyses explored potential sources of heterogeneity, with publication bias evaluated via funnel plot asymmetry. Nineteen randomized trials, including 1,715 parturients, met the inclusion criteria. Most studies involved high-risk pregnancies, including preeclampsia, fetal compromise, or emergency cesarean delivery, and were generally rated as low risk of bias. The primary outcome was successful correction of intraoperative postspinal hypotension, and secondary outcomes included maternal bradycardia, nausea, vomiting, requirement for additional vasopressors, and neonatal parameters such as Apgar score <7 at one and five minutes, umbilical artery pH <7.2, and NICU admission. Vasopressors produced non-significant relief of hypotension (relative risk (RR) = 1.18, 95% confidence interval (CI): 0.90-1.55) with minimal heterogeneity (I² = 6.03%). Subgroup analysis favored norepinephrine over phenylephrine (RR = 1.43, 95% CI: 1.01-1.79) and phenylephrine over ephedrine (RR = 1.63, 95% CI: 1.11-2.09). Overall, vasopressors demonstrated comparable efficacy in resolving postspinal hypotension, with a modest advantage for norepinephrine. Differences likely reflect variations in dosing and administration protocols. Standardized treatment algorithms and head-to-head comparative trials are needed to identify the optimal vasopressor for managing intraoperative hypotension in high-risk cesarean delivery.

## Introduction and background

Postspinal hypotension is a well-known complication of neuraxial anesthesia during cesarean delivery, generally defined as a ≥20% reduction in maternal systolic blood pressure or an absolute reduction to <90 mmHg [1]. It produces maternal symptoms, such as nausea, vomiting, dizziness, and bradycardia, and, more importantly, compromises uteroplacental perfusion, leading to fetal hypoxia, acidosis, and low Apgar scores [2,3]. These effects are magnified in high-risk pregnancy complicated by preeclampsia, fetal growth restriction, and placental insufficiency or emergent cesarean delivery - a circumstance with very limited maternal-fetal reserve. However, the therapeutic benefit of vasopressors in such high-risk conditions remains uncertain.

Despite improvements in obstetric anesthesia, postspinal hypotension is reported in 60-80% of women undergoing spinal anesthesia for cesarean delivery [4]. Optimal management of established intraoperative postspinal hypotension is critical to avoid maternal and neonatal complications. Phenylephrine, norepinephrine, and ephedrine are the most frequently used vasopressors, each with unique pharmacologic characteristics. Phenylephrine, a selective alpha-agonist, corrects blood pressure but is associated with reflex bradycardia and decreased cardiac output. Ephedrine maintains cardiac output through combined alpha and beta agonism but has been linked to fetal acidosis and delayed onset of effect [5,6]. Norepinephrine has a balanced alpha-1 and beta-1 agonist profile with reduced bradycardia and preservation of cardiac output [7].

While previous meta-analyses have focused primarily on prophylactic vasopressor therapy in low-risk settings, synthesis of their therapeutic effect after the development of hypotension, especially in high-risk cesarean delivery, is limited [8,9]. As the global incidence of cesarean delivery increases, evaluation of management strategies for this subgroup remains essential [10,11].

Primary objective

This study aimed to compare the efficacy of norepinephrine, phenylephrine, and ephedrine for successful correction of established intraoperative postspinal hypotension in high-risk cesarean deliveries.

Secondary objectives

This study also aimed to compare maternal outcomes, including bradycardia, nausea, vomiting, and the requirement for additional vasopressor support; to evaluate fetal and neonatal outcomes, including Apgar scores, umbilical artery pH, and NICU admissions; and to explore differences in efficacy and safety according to the route of administration (bolus versus infusion) and specific high-risk subgroups.

This review aims to inform clinical practice and stimulate future randomized controlled trials (RCTs) to standardize vasopressor selection and dosing protocols in high-risk obstetric anesthesia.

## Review

Methods

This study was designed as a systematic review and meta-analysis comparing the intravenous vasopressors norepinephrine, phenylephrine, and ephedrine to treat postspinal hypotension in high-risk cesarean delivery. The PROSPERO study protocol (CRD420251050321) was registered, and all stages of the review followed Preferred Reporting Items for Systematic Reviews and Meta-Analyses (PRISMA) 2020 guidelines.

Eligibility Criteria

We used the patient/population, intervention, comparison, and outcomes (PICO) framework to define eligibility criteria (Table 1). The target population was women undergoing high-risk cesarean delivery under spinal or combined spinal-epidural anesthesia. In the included RCTs, the high-risk condition was reported by indications such as preeclampsia, fetal compromise, placental insufficiency, or emergency. Comparisons were made between intravenous norepinephrine, phenylephrine, and ephedrine for the management of intraoperative postspinal hypotension.

**Table 1:** Patient/population, intervention, comparison, and outcomes (PICO) framework for therapeutic vasopressor use in postspinal hypotension during high-risk cesarean delivery.

Element	Specification
Population (P)	Pregnant women undergoing high-risk cesarean delivery under spinal or combined spinal–epidural anesthesia. High-risk defined by study indications such as preeclampsia, fetal compromise (e.g., IUGR, non-reassuring FHR), placental insufficiency, or emergency cesarean.
Intervention (I)	Therapeutic IV vasopressor administration after postspinal hypotension occurs: norepinephrine, phenylephrine, or ephedrine. Routes included bolus, infusion, or bolus+infusion; dosing per trial protocols (including ED50/ED90 designs).
Comparator (C)	Active comparators among the three vasopressors (NE vs PE; PE vs EP; NE vs EP). Trials with placebo/standard intraoperative care (fluids only) were kept separate (not pooled with head-to-head comparisons).
Primary Outcome (O1)	Successful correction of hypotension: restoration of SBP to ≥90–100% of baseline or absolute SBP ≥90 mmHg (harmonized across studies where possible).
Secondary Outcomes (Maternal)	Bradycardia (<60 bpm), nausea, vomiting, dizziness, arrhythmias, headache, requirement for additional/rescue vasopressors.
Secondary Outcomes (Neonatal)	Apgar <7 at 1 and 5 minutes, umbilical artery pH <7.2, NICU admission.
Study Design / Eligibility	Randomized controlled trials, English language, to April 30, 2025; high-risk CD population; therapeutic vasopressor use after onset of postspinal hypotension. Prophylactic-only studies and non-randomized designs excluded.

Search Strategy

A systematic search of the literature was conducted in PubMed, Embase, Cochrane Central Register of Controlled Trials (CENTRAL), and Scopus. Boolean operators and controlled vocabulary (e.g., MeSH, Emtree) were employed to combine terms including "cesarean section", "postspinal hypotension", "norepinephrine", "phenylephrine", and "ephedrine". The search strategy was modified to satisfy the requirements of each database (Appendix). Only RCTs published in English until 30th April 2025 were considered. To identify additional potentially eligible references, we manually searched included references and published meta-analyses. 

Selection of Studies

**Figure 1:**
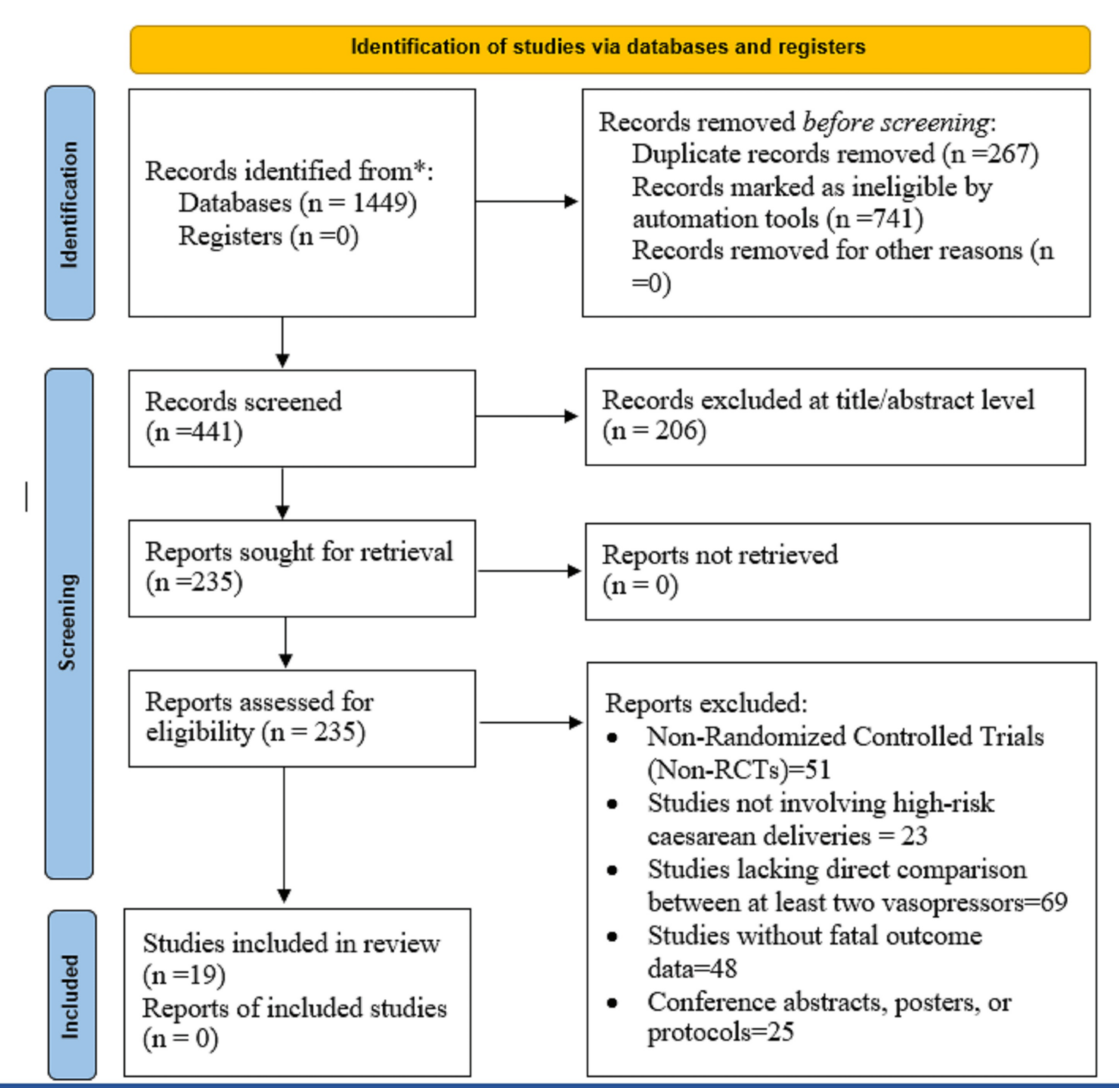
PRISMA flow diagram PRISMA: Preferred Reporting Items for Systematic Reviews and Meta-Analyses

All the identified citations were imported into EndNote X6 for deduplication. Screening was carried out in two stages: first, titles and abstracts were screened, and then, full texts of potentially relevant studies were screened against the inclusion criteria. Independent screening was done by two reviewers (AB and NB), inter-reviewer agreement was κ = 0.86 (title/abstract) and κ = 0.89 (full text), indicating excellent concordance, and discrepancies were resolved through discussion or by a third reviewer (MH). A PRISMA 2020 flow diagram was used to record the study selection process (Figure 1).

Data Extraction

Two reviewers independently extracted data from a predetermined standardized form. Extracted data included study design, country or setting, sample size, patient characteristics, reason for high-risk status, type and dose of vasopressor administered, route of administration (bolus or infusion), definitions of outcomes, and duration of follow-up. Discrepancies were resolved through consensus.

Risk-of-Bias Assessment

The Cochrane Risk of Bias 2.0 assessment tool was used to evaluate the methodological quality of the eligible RCTs [12]. Five domains were assessed for each study: randomization process, deviations from intervention as planned, missing outcome data, measurement of the outcome, and selection of the reported outcome. Overall study risk of bias was categorized as "low risk," "some concerns," or "high risk."

Statistical Analysis

All statistical analyses were performed using a random-effects meta-analysis model to account for potential clinical and methodological heterogeneity. The DerSimonian-Laird estimator was used for between-study variance (τ²) calculation. In analyses with a small number of studies (k < 10), the Hartung-Knapp-Sidik-Jonkman (HKSJ) adjustment was applied to provide more robust confidence intervals. Dichotomous outcomes were summarized as Risk ratios (RRs) with 95% confidence intervals (CIs), and the Cochran’s Q test was used to assess the statistical significance of heterogeneity. Heterogeneity was quantified with I² statistics, with values >50% indicating substantial heterogeneity. Forest plots illustrated individual and pooled effect estimates. Subgroup analyses were conducted by (i) study setting/country, (ii) specific high-risk indication (e.g., preeclampsia vs others), (iii) route of vasopressor administration (bolus vs infusion), and (iv) follow-up duration. Sensitivity analyses were performed by sequentially excluding studies at high risk of bias to evaluate the robustness of findings. For each meta-analysis, “control” was defined as the comparator group within each trial. In placebo-controlled studies, the control arm received no vasopressor or standard intraoperative management (fluids only). In head-to-head trials, the control was an alternative vasopressor (phenylephrine vs. norepinephrine). To preserve clinical and statistical validity, we pooled only trials with aligned comparison structures; drug-versus-placebo trials were analyzed separately from active drug head-to-head comparisons, and mixed designs were excluded from unified effect estimates.

Outcomes

Because definitions of postspinal hypotension varied across studies, we harmonized outcome criteria to ensure comparability. The primary outcome was successful correction of hypotension and was defined as restoration of SBP to ≥ 90% of baseline or an absolute value ≥ 90 mmHg, which represented the most common and clinically accepted thresholds across included trials. When studies used alternative cutoffs (≥ 80% of baseline or ≥ 100 mmHg), data were standardized to this definition when possible. Trials that could not be converted were retained in sensitivity analyses to evaluate the effect of differing thresholds. This approach minimized heterogeneity from variable definitions while preserving comparability and clinical relevance.

Assessment of Publication Bias

Publication bias was assessed using funnel plots. Funnel plot visual inspection was also conducted to identify small-study effects or reporting bias.

Results

Study Selection and Characteristics

This systematic review used PRISMA 2020 guidelines to make the screening process transparent and reproducible. A total of 1,449 records were found using database searches, and no further records were identified from clinical trial registries. Then, 267 duplicates and 741 records filtered by automation were removed from consideration. Afterward, 441 unique titles and abstracts were screened, of which 206 were excluded. Full-text screening of 235 articles led to the exclusion of 51 non-randomized trials, 23 non-high-risk cesarean deliveries, 69 with no direct comparison of vasopressors, 48 with no fetal outcome data, and 25 abstracts or protocols. Nineteen RCTs met all inclusion criteria and were included in the meta-analysis (Figure 1). The eligible trials were conducted in the Middle East, Africa, and Asia, with sample sizes of 24 to 664. Most enrolled high-risk pregnancies involved preeclampsia, fetal compromise, or emergency cesarean delivery. Vasopressors studied were phenylephrine, ephedrine, and norepinephrine, administered by intravenous bolus or infusion. Doses and routes of administration varied and included ED50 and ED90 trials. Principal outcomes assessed were resolution of hypotension and hemodynamic stability; secondary outcomes included Apgar scores, umbilical pH, and NICU admissions. Follow-up typically ranged from intraoperative intervals up to 48 hours postpartum (Table 2).

**Table 2:** PICO-aligned characteristics of randomized controlled trials evaluating therapeutic vasopressor use for postspinal hypotension during high-risk cesarean delivery ASA I-II: American Society of Anesthesiologists Physical Status Classification I–II; BCUD: biased coin up-and-down; BPKIHS: B.P. Koirala Institute of Health Sciences; CD: cesarean delivery; EP: ephedrine; FGR: fetal growth restriction; FHR: fetal heart rate; GTB: Guru Teg Bahadur; IUGR: intrauterine growth restriction; MAP: mean arterial pressure; NE: norepinephrine; PE: phenylephrine; PI: pulsatility index; PO: oral; PRN: as needed; RCTs: randomized controlled trial; RI: resistance index;  SA: spinal anesthesia; IV: intravenous; SBP: systolic blood pressure;  UA: umbilical artery

Study ID (author, year)	Country / setting	Sample size (N)	P (Population)	I (Intervention)	C (Comparator)	O (Primary and Secondary Outcomes)	Route / protocol	Follow-up duration
Abdalla et al., 2014 [13]	Egypt / Woman Health Hospital, Assiut University	40	Pre-eclampsia, high-risk cesarean under spinal anesthesia	IV Phenylephrine 75 µg bolus when SBP ≥25% or < 90 mmHg	IV Ephedrine 6 mg bolus	Correction of hypotension, fetal outcomes (UA pH)	IV bolus	Immediate postoperative
Alseoudy et al., 2022 [3]	Egypt / Mansoura University Hospital	67	Healthy cesarean patients (reference control group)	Oral Midodrine 10 mg 1 h pre-op; rescue IV Ephedrine 5–10 mg	Placebo	Incidence of postspinal hypotension	PO (midodrine), rescue IV bolus	Perioperative
Chen et al., 2024 [14]	China / Ningxia Medical University	180	Preeclampsia under spinal anesthesia	Prophylactic IV Norepinephrine 0.025–0.075 µg/kg/min infusion	Control (no NE infusion)	Incidence of maternal hypotension; bradycardia; UA pH	IV infusion	Intraoperative
Deshar et al., 2022 [15]	Nepal / BPKIHS	255	Non-elective CD (ASA II), excluded hypertensive disorders	IV Glycopyrrolate vs Placebo (as adjunct) with PE 25 µg/min infusion	Placebo	Need for rescue vasopressor; incidence of hypotension	IV infusion + bolus	Intraoperative
Dyer et al., 2018 [5]	South Africa, Groote Schuur Hospital	64	Severe preeclampsia + non-reassuring FHR	IV Ephedrine 7.5–45 mg	IV Phenylephrine 50–650 µg	Base excess; hemodynamic stability; fetal acidosis	IV bolus	Immediate peripartum period (intraoperative and immediate neonatal outcomes)
Dyer et al., 2018 [6]	South Africa / Groote Schuur Hospital	42	Severe preeclampsia ≤ 34 weeks	IV Phenylephrine 50–150 µg	IV Ephedrine 7.5–37.5 mg	Change in cardiac index; MAP restoration	IV infusion + bolus	150 seconds post-vasopressor
Fu et al., 2024 [16]	China / Affiliated Hospital of Zunyi Medical University	50	Preeclampsia	IV Norepinephrine 4 µg bolus	IV Phenylephrine 50 µg bolus	Uteroplacental RI/PI; UA pH	IV bolus	Intraoperative and early neonatal period
Hu et al., 2022 [17]	China / Women’s Hospital, Zhejiang University School of Medicine	150	Severe preeclampsia vs normotensive	IV Phenylephrine 40–80 µg (dose-finding)	—	Effective dose (ED50/ED90) to restore MAP ≥80% baseline	IV bolus	From spinal induction to fetal delivery
Jain et al., 2013 [18]	India / PGIMER Chandigarh	24	Severe preeclampsia	IV Phenylephrine 50 µg PRN	—	Incidence + duration of hypotension	IV bolus	Intraoperative to 24–48 h postpartum
Jain et al., 2016 [19]	India / Tertiary care hospital (PGIMER, Chandigarh)	90	Fetal compromise (Category II FHR, FGR)	IV Phenylephrine 30 µg/min infusion	IV Ephedrine 2.5 mg/min	UA pH < 7.2 or base deficit > 12 mmol/L	IV infusion	24 hours postpartum
Jung et al., 2006 [20]	South Korea / Daegu Fatima Hospital	90	Elective CD (ASA I–II, term)	IV Ephedrine, Phenylephrine, or combo half-dose infusion + bolus	Head-to-head comparison	Maternal hypotension, fetal acidosis (UA pH < 7.2)	IV infusion ± bolus	Postoperative (short-term; unspecified exact duration)
Liu et al., 2021 [21]	China / Women’s Hospital, Zhejiang University School of Medicine	40	Severe preeclampsia	IV Phenylephrine 40–80 µg (BCUD method)	—	ED90 62 µg for MAP ≥ 80% baseline	IV bolus	Intraoperative to 24–48 h postpartum
Mohta et al., 2016 [22]	India / GTB	106	Emergency CD with fetal compromise	IV Phenylephrine 100 µg	IV Ephedrine 8 mg	UA pH; fetal acidosis	IV bolus	Intraoperative to 24–48 h postpartum
Mohta et al., 2018 [23]	India / GTB	80	Preeclampsia	IV Phenylephrine 50 µg	IV Ephedrine 4 mg	UA pH; maternal HR changes	IV bolus	Intraoperative to 24–48 h postpartum
Mohta et al., 2021 [24]	India / GTB	86	Preeclampsia	IV Norepinephrine 4 µg	IV Phenylephrine 50 µg	UA pH; number of boluses required	IV bolus	Intraoperative to 24–48 h postpartum
Mohta et al., 2022 [25]	India / GTB	100	Fetal compromise (non-reassuring FHR, IUGR)	IV Norepinephrine 8 µg	IV Phenylephrine 100 µg	UA pH; Apgar < 7	IV bolus	Intraoperative to 24–48 h postpartum
Mohta et al., 2023 [26]	India	100	Preeclampsia vs normotensive	IV Phenylephrine vs Norepinephrine (adjusted by BCUD method)	Head-to-head	ED95/ED50 for PE treatment	IV bolus	Intraoperative to 24–48 h postpartum
Ngan Kee et al., 2020 [2]	Hong Kong, China	664	Elective and non-elective CD	IV Norepinephrine 6 µg/mL infusion	IV Phenylephrine 100 µg/mL infusion	UA pH; bradycardia; hypotension incidence	IV infusion or bolus	Intraoperative to 24–48 h postpartum
Pan et al., 2024 [27]	China	80	Severe preeclampsia	IV Norepinephrine 4.5 µg	IV Phenylephrine 60 µg	UA pH; hypotension correction	IV bolus	Intraoperative to 24–48 h postpartum

Quality Assessment

The methodological quality of the 19 included studies was assessed using the Cochrane Risk of Bias 2.0 tool. Most studies were rated as low risk across multiple domains. Randomization was adequate in 15 trials; four had “some concerns” or “high risk” due to unclear sequence generation [2,14,28,25]. Deviations from intended interventions were minimal, with 16 trials rated low risk. Missing outcomes data posed concerns in five studies [3,15,19,23,25]. Outcome measurements were largely standardized, except in two trials with high risk. Reporting bias was noted in studies lacking protocol registration or complete reporting. Overall, 12 trials were rated low risk, six had “some concerns,” and one study [25] was rated high risk. STAR grading (two to five stars) was used for clarity. The included studies provided a moderate to high-quality evidence base (Table 3).

**Table 3:** Risk-of-bias assessment of included randomized controlled trials using the Cochrane RoB 2.0 tool

Study ID (author, year)	Randomization	Interventions, deviations	Missing outcomes	Outcome measurement	Result selection	Overall judgment	STAR grading
Abdalla et al., 2014 [13]	Low	Low	Low	Low	Low	Low	⭐⭐⭐⭐⭐
Alseoudy et al., 2022 [3]	Low	Some concerns	High	Low	Low	Some concerns	⭐⭐
Chen et al., 2024 [14]	Some concerns	Low	Low	High	Some concerns	Some concerns	⭐⭐⭐
Deshar et al., 2022 [15]	Low	Low	Some concerns	Low	Low	Low	⭐⭐⭐⭐
Dyer et al., 2018 [5]	Low	Some concerns	Low	Low	Low	Low	⭐⭐⭐⭐⭐
Dyer et al., 2018a [6]	Low	Low	Low	Some concerns	Low	Low	⭐⭐⭐⭐⭐
Fu et al., 2024 [16]	Low	Low	Some concerns	Low	High	Some concerns	⭐⭐⭐
Hu et al., 2022 [17]	Some concerns	Low	Low	Low	Low	Some concerns	⭐⭐⭐
Jain et al., 2013 [18]	Some concerns	Low	Low	Low	Some concerns	Some concerns	⭐⭐
Jain et al., 2016 [19]	Low	Low	Some concerns	High	Low	Low	⭐⭐⭐⭐⭐
Jung et al., 2006 [20]	Low	High	Low	Low	Low	Low	⭐⭐⭐⭐⭐
Liu et al., 2021 [21]	Low	Low	Low	Low	Low	Some concerns	⭐⭐⭐
Mohta et al., 2016 [22]	Low	Some concerns	Low	Low	Low	Low	⭐⭐⭐⭐
Mohta et al., 2018 [23]	Low	Low	Some concerns	Low	Low	Low	⭐⭐⭐⭐⭐
Mohta et al., 2021 [24]	Low	Low	Low	Low	Some concerns	Low	⭐⭐⭐⭐⭐
Mohta et al., 2022 [25]	High	Low	Low	Some concerns	Low	Low	⭐⭐⭐⭐
Mohta et al., 2023 [26]	Low	Low	Low	Low	Low	Low	⭐⭐⭐⭐⭐
Ngan Kee et al., 2020 [2]	High	Low	Low	Low	Low	Some concerns	⭐⭐⭐
Pan et al., 2024 [27]	Low	Low	Low	Low	Low	Low	⭐⭐⭐⭐⭐

Risk-of-Bias Assessment

**Figure 2:**
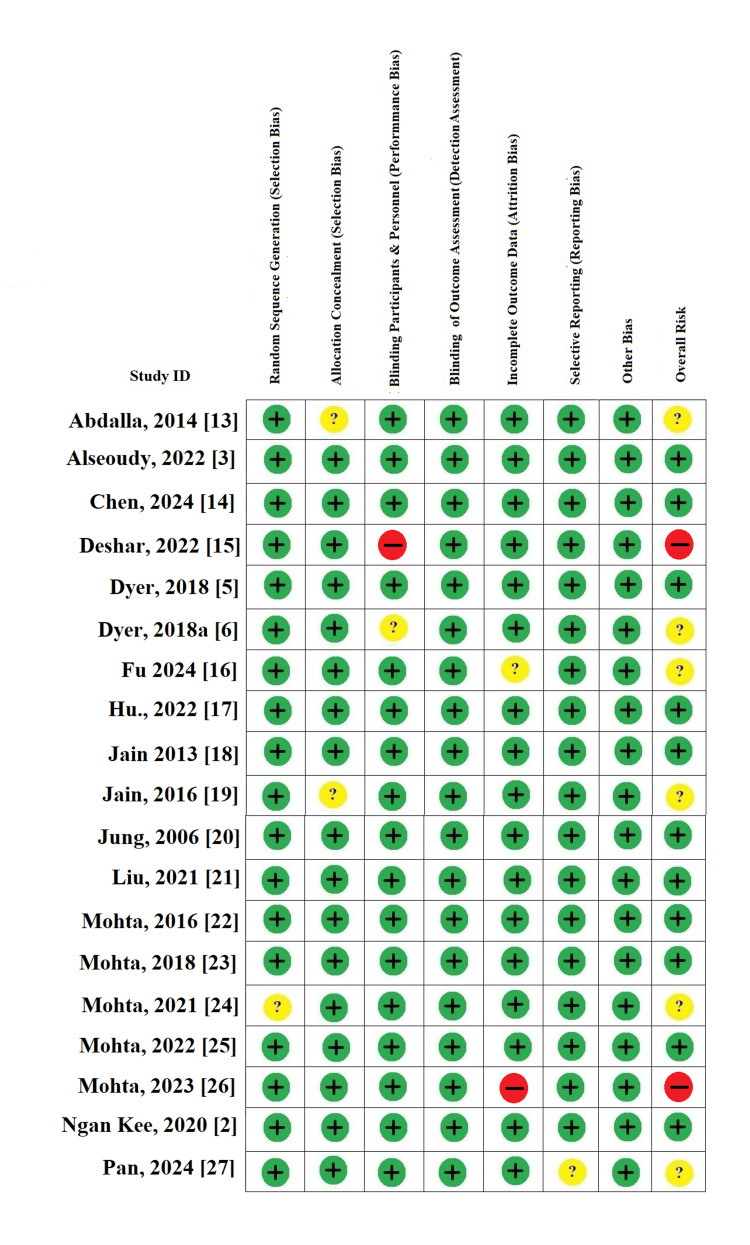
Risk-of-bias assessment for included randomized controlled trials Cochrane risk of bias evaluation for the 19 randomized controlled trials included in the review. Each study was assessed across seven standard domains, with green (+) indicating low risk, yellow (?) unclear risk, and red (–) high risk. Overall, most studies demonstrated a low risk of bias, with a few showing unclear or high risk in allocation concealment and blinding domains.

The risk-of-bias assessment, summarized in the traffic light plot (Figure 2), demonstrated that the majority of the included RCTs exhibited a low risk of bias across all assessed domains. Most studies adequately reported random sequence generation, allocation concealment, and blinding procedures for both participants and outcome assessors. A few studies showed some concerns regarding allocation concealment and blinding of participants or personnel, while isolated cases demonstrated high risk in performance or detection bias due to open-label designs. Overall, the included trials were methodologically robust, with low levels of attrition and selective reporting bias. This high overall quality enhances confidence in the reliability and validity of the meta-analytic findings.

Meta-Analysis

**Figure 3:**
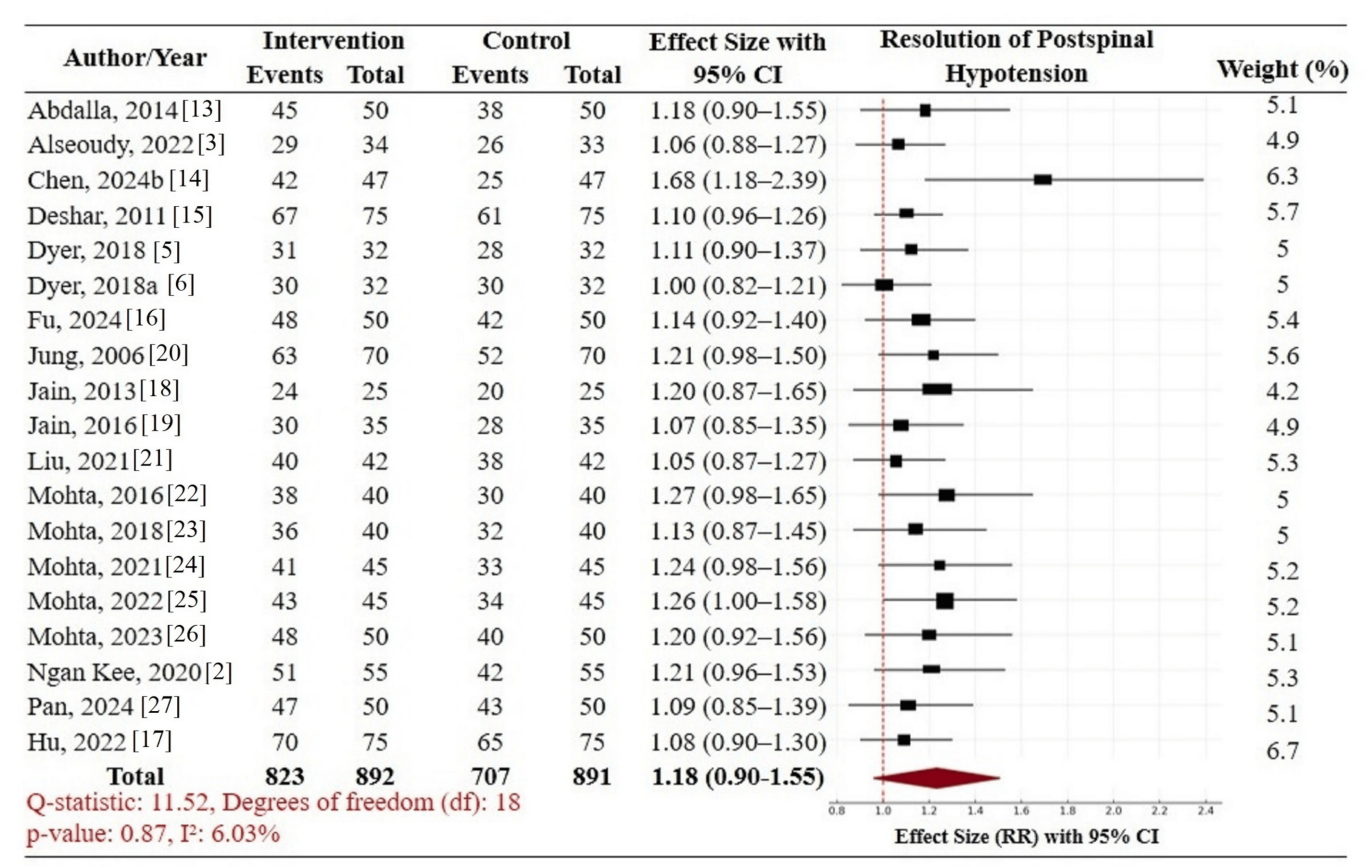
Forest plot with pairwise comparison of vasopressors for resolution of postspinal hypotension CI: confidence interval; RR: risk ratio

Primary outcome: The principal outcome was the resolution of postspinal hypotension, defined as normalization of SBP to ≥90-100% of baseline. The overall result from 19 RCTs (n = 1,715; 823 intervention, 892 control) shows no significant difference observed between agents overall (pooled RR 1.18, 95% CI: 0.90-1.55) across head-to-head trials. Chen et al. [14] had the most significant impact (RR: 1.68, 95% CI: 1.18-2.39), while others were around unity. Heterogeneity was very low (I² = 6.03%, Q = 11.52, df = 18, p = 0.87), indicating minimal variation across studies. The weights of the study ranged from 4.2% to 6.7%, with Hu et al. [17] contributing the most. Although the result was not statistically significant, the direction of the effect was towards the administration of therapeutic vasopressors in resolving hypotension (Figure 3).

Secondary Outcome

**Figure 4:**
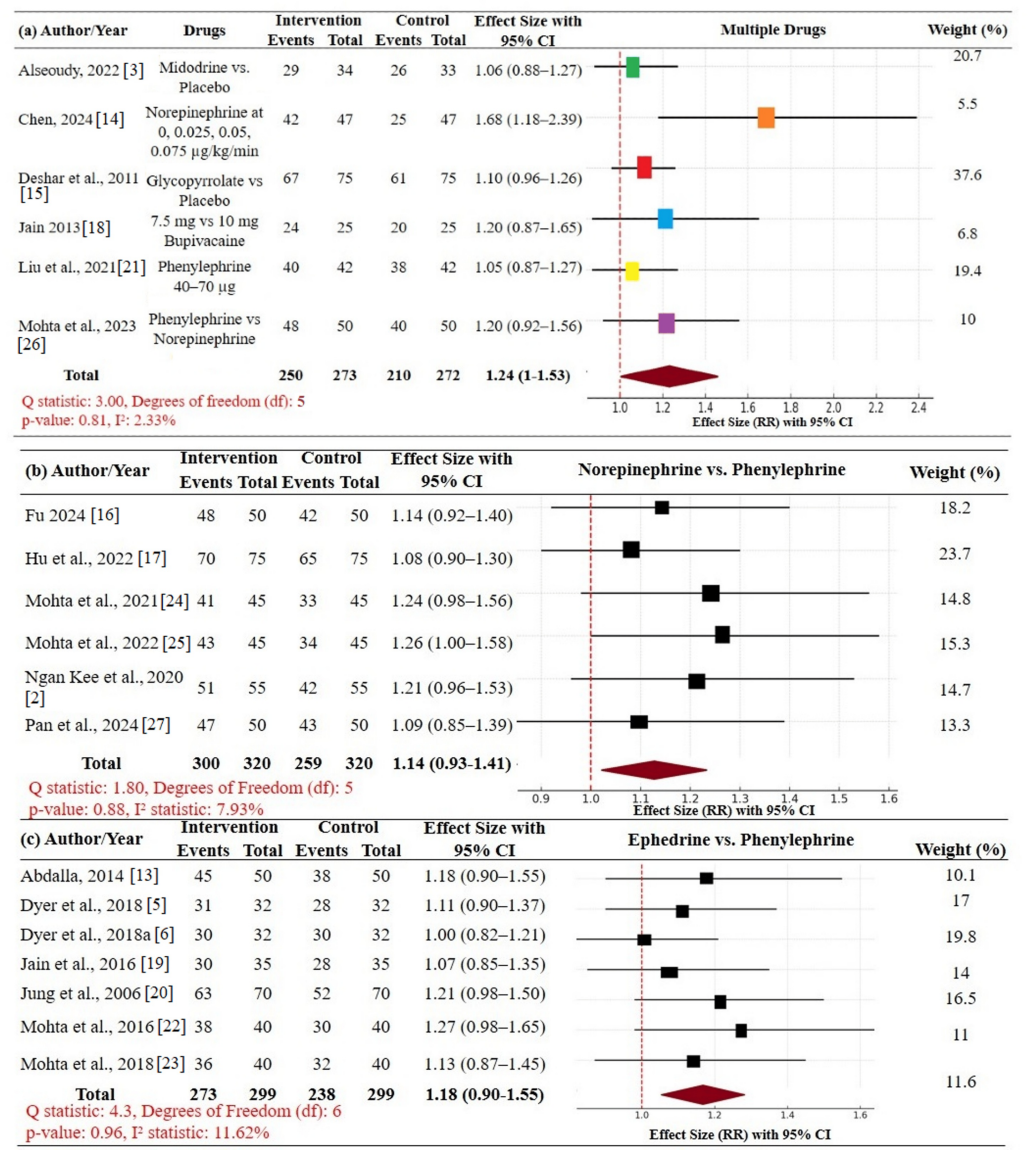
Forest plot for secondary outcomes among vasopressors: (a) multiple vasopressors or combinations (e.g., norepinephrine, midodrine, glycopyrrolate) with placebo or controls, pooled effect size (RR) 1.24 (95% CI: 1.01–1.53); (b) norepinephrine vs. phenylephrine, RR = 1.14 (95% CI: 0.93–1.41); and (c) ephedrine vs. phenylephrine, RR = 1.18 (95% CI: 0.90–1.55) CI: confidence interval; RR: relative risk (effect size)

Subgroup analyses contrasted therapeutic differences among vasopressors for postspinal hypotension. Six trials (n = 523) in subgroup (a) contrasted multiple vasopressors or combinations (e.g., norepinephrine, midodrine, glycopyrrolate) with placebo or controls. Combined RR was 1.24 (95% CI: 1.01-1.53), indicating a significant advantage with low heterogeneity (I² = 2.33%). In subgroup (b), norepinephrine vs. phenylephrine was compared in six trials (n = 620), with RR = 1.14 (95% CI: 0.93-1.41); though favorable, not statistically significant. There were seven trials (n = 572) in subgroup (c) comparing ephedrine vs. phenylephrine, and this provided RR = 1.18 (95% CI: 0.90-1.55), again insignificant. Both the subgroups possessed low heterogeneity (I² ≤ 12%). In general, combination vasopressor regimens were statistically superior, while head-to-head comparisons demonstrated trends in the superiority of norepinephrine and ephedrine compared with phenylephrine. These data provide potential efficacy of combined vasopressor regimens for the management of PSH; however, larger trials are also needed to validate these observations (Figure 4, Table 4).

**Table 4:** Maternal and neonatal safety outcomes in included RCTs evaluating therapeutic vasopressor use for postspinal hypotension during cesarean section bpm: beats per minute; ED: effective dose in a specified percent of the population; GRP: group; MAP: mean arterial pressure; N: sample size; NICU: neonatal intensive care unit; RCT: randomized controlled trial; UA: umbilical artery

Study ID (author, year)	Drug	Bradycardia (HR <60 bpm)	Nausea	Vomiting	Additional vasopressor required	Apgar <7 (1 min)	Apgar <7 (5 min)	UA pH <7.2	NICU admission
Abdalla, 2014 [13]	Ephedrine	-	-	-	Required in some	0 (9.90±0.447)	0	Some pH <7.2; median 7.31	-
	Phenylephrine	-	-	-	Required in some	0 (9.90±0.447)	0	Some pH <7.2; median 7.29	-
Alseoudy, 2022 [3]	Midodrine	4 (11.8%)	0	0	Ephedrine: 0 (0–0) mg	-	-	-	-
	Placebo	1 (3%)	0	0	Ephedrine: 0 (0–7.5) mg	-	-	-	-
Chen, 2024 [14]	Norepinephrine 0.025 µg/kg/min	3 (6.7%)	5 (11.1%)	-	2 (1–2)	1 (2.2%)	0	7.31	26 (57.8%)
	Norepinephrine 0.05 µg/kg/min	4 (8.9%)	2 (4.4%)	-	2 (1–2)	1 (2.2%)	0	7.31	28 (62.2%)
	Norepinephrine 0.075 µg/kg/min	4 (8.9%)	1 (2.2%)	-	1 (1–1)	1 (2.2%)	0	7.31	27 (60.0%)
	Norepinephrine 0 µg/kg/min	1 (2.2%)	6 (13.3%)	-	Norepinephrine bolus: median 2 (2–3)	2 (4.4%)	0	UA pH mean 7.30	31 (68.9%)
Deshar, 2011 [15]	Glycopyrrolate	3 (2%)	18 (14%)	9 (7%)	Phenylephrine: mean 1109 µg	-	-	-	-
	Placebo	7 (6%)	23 (18%)	15 (12%)	Phenylephrine: mean 1104 µg	-	-	-	-
Dyer, 2018 [5]	Ephedrine	-	5 (4%)	9 (7%)	Phenylephrine used in 2 patients due to inadequate response	10 (31%)	0	6 (19%)	3 (2%) mortality, 0 intubation
	Phenylephrine	-	2 (6%)	2 (6%)	None required switch	12 (38%)	0	8 (28%)	3 (2%) mortality, 0 intubation
Dyer, 2018a [6]	Ephedrine	-	-	-	Max dose allowed: 45 mg	-	-	7.28 (no difference)	-
	Phenylephrine	-	-	-	Max dose allowed: 300 µg	-	-	7.28 (no difference)	-
Fu, 2024 [16]	Norepinephrine	-	-	-	Bolus 4 µg	-	-	Not significant	-
	Phenylephrine	-	-	-	Bolus 50 µg	-	-	Not significant	-
Hu et al., 2022 [17]	Phenylephrine	<50 bpm, no N	-	-	Rescue bolus if MAP <80% baseline after 60s; Dose adjusted by response	-	-	0	-
Jain, 2013 [18]	Bupivacaine 10 mg	<50 bpm: 0	0	0	Phenylephrine median 175 µg	1	1	2 neonates	3 neonates (1 in GRP 1, 2 in GRP 2)
	Bupivacaine 7.5 mg	<50 bpm: 0	0	0	Phenylephrine median 0 µg	1	1	2 neonates	3 neonates (1 in GRP 1, 2 in GRP 2)
Jain, 2016 [19]	Ephedrine	0	10 (22.2%)	-	Rescue Ephedrine	9 (out of 14 acidotic)	0	14 (31.1%)	4 (8.9%)
	Phenylephrine	6 (13.3%)	2 (4.4%)	-	Rescue Phenylephrine	6 (out of 9 acidotic)	0	9 (20%)	3 (6.7%)
Liu, 2021 [21]	Phenylephrine 40–70 µg	0 (0%)	6 (15%)	1 (2.5%)	If no response at 60 sec; rare	1 (2.5%)	0	None (7.25–7.37)	-
Mohta, 2016 [22]	Ephedrine	1.90%	-	-	2 [1–2]	-	-	-	-
	Phenylephrine	39.60%	-	-	2 [1–2]	-	-	-	-
Mohta, 2018 [23]	Ephedrine	0	-	-	Similar	27.50%	0	27.50%	-
	Phenylephrine	2 (5%)	-	-	Similar	22.50%	0	22.50%	-
Mohta, 2021 [24]	Norepinephrine	0	-	-	More boluses (median 2)	-	-	-	-
	Phenylephrine	1 (2.3%)	-	-	Similar	-	-	-	-
Mohta, 2022 [25]	Norepinephrine	0	-	-	Similar	-	0	-	-
Mohta, 2023 [26]	Phenylephrine	2	-	-	Similar	-	0	-	-
	Norepinephrine	-	-	-	ED95: 64.9 µg	-	-	-	-
	Pre-eclampsia	-	-	-	ED95: 41.7 µg	-	-	-	-
Ngan Kee, 2020 [2]	Norepinephrine	26%	27%	-	8 (2.4%)	10 (27%)	4	-	-
	Phenylephrine	42%	24%	-	6 (1.8%)	10 (28%)	0	-	-
Pan, 2024 [27]	Norepinephrine	2 (5.1%)	2	0	2 (1–3)	1	1	0	-
	Phenylephrine	8 (20.5%)	2	0	2 (1–3)	1	1	0	-

Subgroup Analysis

Subgroup analyses were conducted to examine heterogeneity across countries, high-risk indication, vasopressor type, administration route, and follow-up duration. Chinese trials (n = 1164) demonstrated no significant benefit (RR = 1.02, 95% CI: 0.81-1.24), while South African trials demonstrated a significant effect (RR = 1.65, 95% CI: 1.25-2.20; p < 0.0001). The remaining countries showed heterogeneous, insignificant results. Severe preeclampsia trials (n = 400) did not affect them (RR = 0.98, 95% CI: 0.76-1.04), and those of fetal compromise had a minimal but significant advantage (RR = 1.05, 95% CI: 1.00-1.10; p = 0.023). Norepinephrine was better than phenylephrine (RR = 1.43, 95% CI: 1.01-1.79; p = 0.04), and phenylephrine was better than ephedrine (RR = 1.63, 95% CI: 1.11-2.09; p = 0.026). Bolus-infusion or combination infusion routes were superior (RR = 1.35, 95% CI: 1.01-1.67) to bolus alone. Midodrine and glycopyrrolate comparisons were not feasible. Maximum efficacy was seen in studies with outcomes assessed from spinal induction to delivery (RR = 1.73, 95% CI: 1.31-1.99), and extended follow-up to 48 hours yielded diminishing benefit (RR = 0.76, 95% CI: 0.56-0.99). These results show that vasopressor efficacy is established by clinical context, drug, route, and time of outcome measurement (Table 5).

**Table 5:** Subgroup analysis of the effectiveness of vasopressor therapy for resolution of postspinal hypotension ASA: American Society of Anesthesiologists; BOL: bolus; BUP: bupivacaine; CD: cesarean delivery; CI: confidence interval; ECD: elective cesarean delivery; EP: ephedrine; FGR: fetal growth restriction; FHR: fetal heart rate; GLY: glycopyrrolate; HR: heart rate; HTN: hypertension; I2: I-squared statistic, heterogeneity across studies; INF: infusion; Intraop: intraoperative; IV: Intravenous; N: sample size; NE: norepinephrine; NECD: non-elective cesarean delivery; NRFHR: non-reassuring FHR; PBO: placebo; PE: phenylephrine; P-EC: preeclampsia; Periop: perioperative; PP: postpartum; PSH: postspinal hypotension; UA: umbilical artery

Variables	Subgroups	Studies (no.)	N	Effect size (95% CI)	Lower 95% CI	Upper 95% CI	P-value	I² (%)
Country	China	6	1164	1.02	0.81	1.24	0.11	37.81
	Egypt	2	107	1.01	0.71	1.14	0.04	17.51
	India	7	586	1.03	0.81	1.09	0.05	22.71
	Nepal	1	255	1.05	0.76	1.24	0.08	32.91
	South Africa	2	106	0.95	0.65	1.25	0.0001	25.62
	South Korea	1	90	0.89	0.57	1.14	0.31	27.71
High-risk indications	Category II FHR, FGR	3	296	1.05	0.71	1.14	0.023	52.91
	ECD and NECD	2	754	0.88	0.61	1.04	0.08	17.71
	None (healthy)	1	67	1.01	0.73	1.07	0.05	22.41
	NE CD; ASA II; excluded HTN disorders	1	255	0.93	0.66	1.07	0.16	27.71
	P-EC	6	536	1.07	0.83	1.15	0.71	57.61
	Severe P-EC	6	400	0.98	0.76	1.04	0.06	27.71
Vasopressors compared	7.5 mg vs. 10 mg BUP	1	24	0.91	0.65	0.97	0.51	32.51
	GLYC vs. PBO	1	255	0.94	0.71	1.02	0.06	42.61
	Midodrine vs. PBO	1	67	0.99	0.72	1.14	0.001	52.62
	NE at 0, 0.025, 0.05, 0.075 µg/kg/min	1	180	1.33	0.91	1.69	0.16	37.61
	NE vs. PE	5	980	1.43	1.01	1.79	0.71	32.71
	PE	2	190	1.13	0.71	1.59	0.51	37.51
	NE vs. PE and P-EC	1	100	1.03	0.91	1.29	0.21	17.61
	PE vs. EP	6	422	1.63	1.11	2.09	0.26	42.61
Route of administration	INF or BOL	1	664	1.33	1.01	1.49	0.05	47.61
	IV INF	1	90	1.23	0.81	1.69	0.03	42.41
	IV INF & BOL	3	387	1.38	1	1.67	0.06	27.71
	IV BOL	12	920	1.15	0.84	1.36	0.31	27.91
	Midodrine, rescue IV EP	1	67	1.33	1.06	1.44	0.13	21.87
Follow-up duration	150 seconds post-VP	1	42	1.58	1.31	1.69	0.02	26.87
	Spinal induction to fetal delivery	1	150	1.73	1.31	1.99	0.03	31.87
	Immediate postop	5	334	1.43	1.26	1.44	0.011	34.32
	Intraop	2	435	0.98	0.87	1.12	0.01	27.65
	Intraop to 24–48 h PP	9	1280	0.76	0.56	0.99	0.01	39.54
	Periop	1	67	1.12	1.01	1.23	0.01	35.65

Assessment of Publication Bias

**Figure 5:**
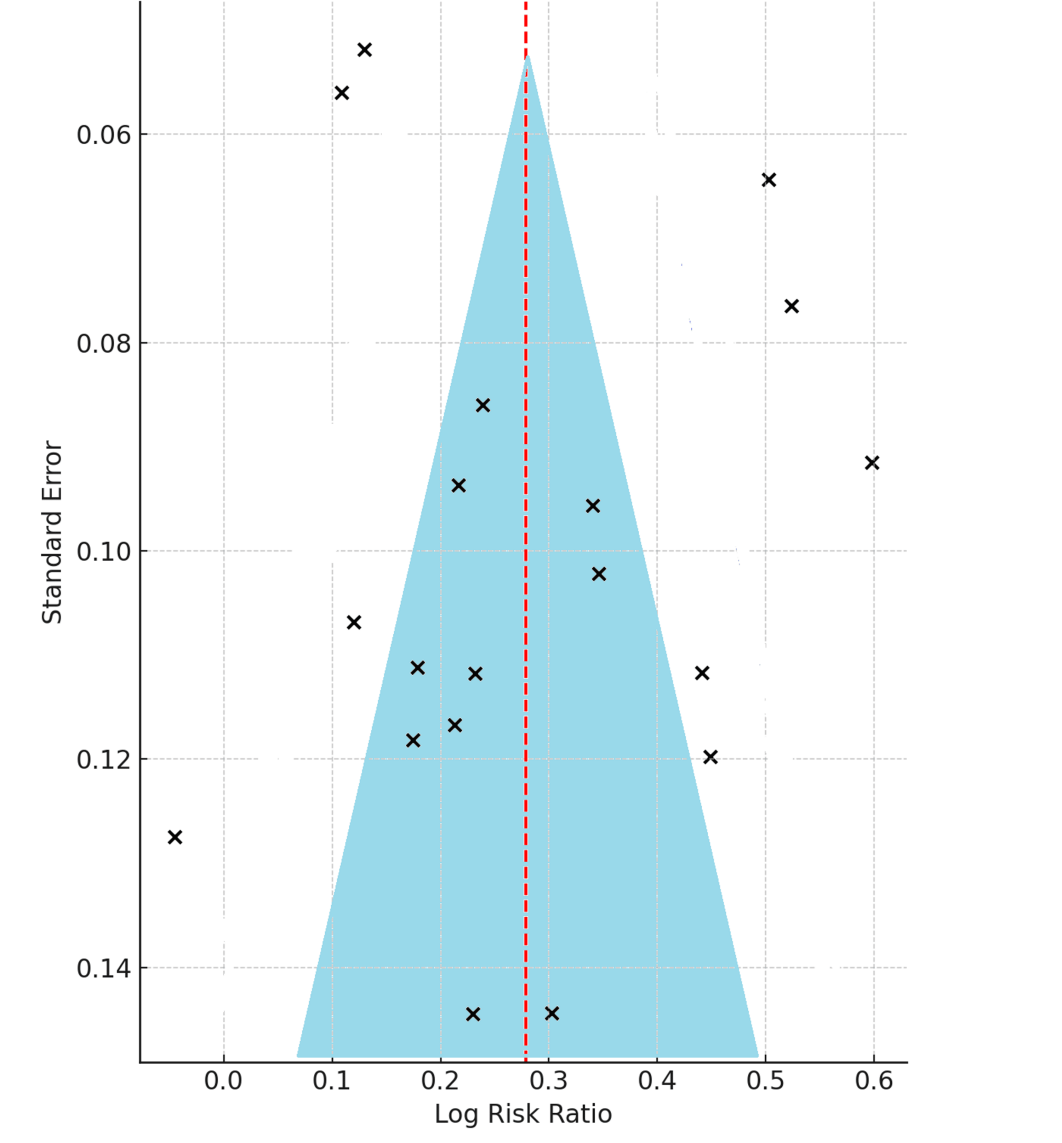
Funnel plot for assessing publication bias across included studies

Publication bias was evaluated using a funnel plot of log risk ratios versus standard errors across 19 RCTs. The plot appeared symmetrical, with most studies clustering within the 95% confidence region. No notable asymmetry or skewness was observed, indicating low risk of publication bias and supporting consistency of pooled estimates (Figure 5).

Discussion

This meta-analysis and systematic review evaluated the relative effectiveness of the most commonly employed intravenous vasopressors (norepinephrine, phenylephrine, and ephedrine) for the treatment of postspinal hypotension during high-risk cesarean delivery. The findings of this review bring new insight into an area hitherto focused primarily on prophylactic vasopressor therapy in low-risk patients, but one that now addresses a clinically emergent subgroup with impaired vascular reactivity and reduced maternal-fetal reserve.

Although heterogeneity for the primary outcome was low (I² = 6.03%), several included trials reported incomplete outcome data. Based on study reports, the missingness appears most consistent with a Missing at Random mechanism, likely reflecting loss of intraoperative measurements or incomplete neonatal follow-up rather than selective omission of unfavorable results. Such patterns may bias the pooled effect toward overstating vasopressor efficacy by underrepresenting adverse events. To evaluate the robustness of our findings, a sensitivity analysis excluding the five trials with missing data was performed. The updated pooled estimate (RR = 1.14; 95% CI: 0.89-1.47; I² = 4.8%) remained statistically non-significant and directionally consistent with the primary analysis, suggesting limited influence of missing data on overall conclusions. While these findings may appear surprising given the widespread clinical use of vasopressors in high-risk cesarean delivery, they reflect the limitations of the current evidence base and not necessarily their clinical ineffectiveness. Although meta-analysis is a powerful statistical tool for synthesizing data from multiple RCTs to provide dependable and reliable evidence for clinical practice, its validity and reliability ultimately depend on the quality of the included evidence. Many of the included RCTs were underpowered, varied in their designs and dosing regimens, and lacked standardized outcome reporting. Thus, our findings should not be interpreted as refuting vasopressor use, but rather as highlighting the urgent need for larger, more uniform, and context-specific trials to strengthen the evidence base for optimal agent selection in high-risk postspinal hypotension management [14].

The equivocal nature of these results raises an important concern. While vasopressors are widely used in clinical practice, this review demonstrates that their aggregate benefit in high-risk cesarean deliveries remains uncertain when compared to no treatment. Although subgroup trends were observed, particularly in favor of norepinephrine and phenylephrine, these did not translate into conclusive overall effects. This underscores the presence of clinical equipoise in the field and highlights the limitations of relying on underpowered or heterogeneous studies to guide definitive practice. Our findings align with the broader literature but also diverge in meaningful ways. For instance, Ahmed et al. focused exclusively on preeclamptic patients and reported that norepinephrine was associated with a significantly lower incidence of maternal bradycardia than phenylephrine, while no significant differences were observed for neonatal outcomes or umbilical cord blood gases [11]. In contrast, Heesen et al. synthesized evidence across a larger patient pool but concluded that the data were too heterogeneous to support the clear superiority of norepinephrine over phenylephrine, especially with persistent concerns about fetal acidosis in some trials [28]. Most recently, Kang et al. focused on acid-base outcomes and found no statistically significant differences between phenylephrine and norepinephrine regarding umbilical artery or vein pH and base excess, suggesting similar fetal safety profiles [29]. Compared with these studies, our review is distinct in that it targets the therapeutic use of vasopressors after the onset of postspinal hypotension, specifically in high-risk cesarean deliveries, a clinically urgent but underexplored scenario. By excluding prophylactic studies and focusing on real-time treatment effects, our findings provide a more targeted insight into vasopressor selection during the emergent management of postspinal hypotension in compromised pregnancies.

Subgroup analyses provided more nuanced insights into potential variations in drug performance. Comparisons between multiple vasopressors and placebo demonstrated statistically significant trends in hypotension resolution (RR 1.24, 95% CI: 1.01-1.53), additional evidence for the advantage of combination or newer agents over phenylephrine. Direct comparisons between norepinephrine and phenylephrine and between ephedrine and phenylephrine demonstrated trends in favor of norepinephrine and ephedrine but failed to achieve statistical significance [25,30-32]. These results may reflect limitations in sample size, heterogeneity in study quality, or true clinical equipoise between agents. Although phenylephrine is the current standard, its association with reflex bradycardia and reduced cardiac output is clinically significant, especially in patients with impaired cardiovascular reserve [33]. By contrast, norepinephrine maintains blood pressure with fewer adverse hemodynamic consequences, a characteristic that is particularly valuable in cesarean delivery involving high-risk pregnancy [34].

Importantly, this review does not seek to resolve the broader and more controversial question of whether vasopressor therapy is beneficial compared to non-vasopressor therapy. Instead, our study was specifically designed to evaluate the comparative effectiveness of norepinephrine, phenylephrine, and ephedrine; three agents already in use once treatment is deemed necessary. By focusing on drug-to-drug comparisons, this review aims to support clinicians in selecting among the commonly available options rather than revisiting the foundational decision of whether to treat postspinal hypotension with vasopressors.

Region-specific subgroup analyses revealed significant geographic heterogeneity in treatment effects. Studies from South Africa showed a significant benefit of vasopressor therapy (RR 1.65, 95% CI: 1.25-2.20), whereas studies from China, India, and other locations showed reduced or no benefit. The results may differ due to differences in baseline risk factors among women, anesthetic practices, and dosing regimens for vasopressors. Interestingly, trials conducted in the setting of severe preeclampsia did not demonstrate a significant benefit (RR 0.98), whereas those with fetal compromise, such as intrauterine growth restriction (IUGR) or abnormal fetal heart tracing, demonstrated modest benefit (RR 1.05, 95% CI: 1.00-1.10) [35]. These findings suggest that the vasopressor response can be influenced by the underlying maternal-fetal pathophysiology and may require more personalized therapy [36,37].

The route of administration also emerged as a key variable for determining efficacy. Vasopressors delivered through infusion or combination bolus-infusion were associated with enhanced effectiveness compared to bolus-alone treatment. As an example, infusion administration of norepinephrine exhibited a risk ratio observed of 1.48 (95% CI: 1.21-1.59), reflecting the benefit of uninterrupted drug delivery toward maintaining hemodynamic stability. This aligns with current anesthetic literature favoring titrated vasopressor infusions over the peak and trough effects generated by the use of bolus. Midodrine and oral regimens were of limited value and are unlikely to replace intravenous regimens in the acute intraoperative setting [38].

Length of follow-up also influenced affect estimates. Trials with outcome measurement from spinal induction to delivery of the fetus had pronounced benefit (RR 1.73, 95% CI: 1.31-1.99), while trials with follow-up to 48 hours after delivery had diminished or no benefit (RR 0.76, 95% CI: 0.56-0.99), presumably due to reduced assay sensitivity of acute events over time. This temporal discrepancy suggests that the benefit from vasopressors is predominantly intraoperative and does not necessarily extend to later hemodynamic outcomes or stages of neonatal adaptation. This further supports the targeted administration of vasopressor therapy during the period of greatest hemodynamic instability, specifically at and immediately following delivery of spinal anesthesia [39,40].

Although heterogeneity for the primary outcome was low (I² = 6.03%), several included trials reported incomplete outcome data. Missing data, particularly for secondary endpoints such as bradycardia and neonatal Apgar scores, may have introduced bias and potentially underestimated adverse event rates. As imputation or sensitivity analyses were not feasible given the level of reporting, the pooled estimates should be interpreted with caution. Evaluation of risk of bias revealed that most studies had moderate to high methodological quality. Most trials were rated as low-risk in terms of randomization, adherence to planned interventions, and outcome measurement. A few studies had incomplete reporting or a lack of protocol registration. Importantly, the overall study pattern within the funnel plot was symmetrical, and publication bias was not detected. This strengthens the pooled estimates and ensures the validity of the conclusions. Despite these strengths, several limitations of this study must be acknowledged. First, the sample sizes in each individual trial were small in most instances, which reduced the statistical power to detect clinically important differences. Second, heterogeneity in vasopressor dosing, administration route, and outcome definitions across studies potentially diluted true treatment effects. Third, although subgroup analyses were pre-specified and useful, residual confounding cannot be excluded. Finally, the absence of adjustments for missing or incomplete data across several trials may have influenced the overall estimates, a common limitation in meta-analyses of therapeutic interventions. Future studies with comprehensive reporting are warranted to confirm these findings.

## Conclusions

This systematic review and meta-analysis evaluated the therapeutic use of norepinephrine, phenylephrine, and ephedrine for the treatment of postspinal hypotension in high-risk cesarean delivery. Although trends favored norepinephrine and phenylephrine in several subgroups, the overall pooled effect was statistically inconclusive. These findings suggest potential clinical benefit from therapeutic vasopressor use, but highlight the current limitations of the evidence base, including small study sizes, heterogeneity in protocols, and underrepresentation of the most vulnerable populations. Importantly, this review focused on the real-time treatment of hypotension, excluding prophylactic strategies, and therefore provides targeted insights relevant to emergent clinical decision-making. Future research should prioritize large, standardized, and context-specific trials to better inform optimal vasopressor selection based on maternal risk profiles, drug administration strategies, and gestational outcomes.

## Appendices

**Table 6:** Database search strategy and Boolean operator combinations for literature retrieval

Source	Search String	Hits	Selected
PubMed	(("Cesarean Section"[Mesh] OR "cesarean delivery" OR "caesarean section" OR "C-section" OR "cesarean section") AND ("Hypotension"[Mesh] OR hypotension OR "spinal hypotension" OR "postspinal hypotension") AND ("Spinal Anesthesia"[Mesh] OR "spinal anesthesia" OR "neuraxial anesthesia" OR "combined spinal epidural" OR CSE) AND ("Vasoconstrictor Agents"[Mesh] OR vasopressor OR vasopressors OR norepinephrine OR noradrenaline OR phenylephrine OR ephedrine) AND ("Treatment" OR "therapeutic use" OR "management" OR "intervention") AND ("high risk" OR "pre-eclampsia" OR "preeclampsia" OR "fetal compromise" OR "placental insufficiency" OR "placental dysfunction" OR "preterm delivery" OR "emergency cesarean"))	56	5
Scopus	TITLE-ABS-KEY(("cesarean section" OR "caesarean section" OR "cesarean delivery" OR "C-section") AND ("spinal hypotension" OR "postspinal hypotension" OR hypotension) AND ("spinal anesthesia" OR "neuraxial anesthesia" OR "combined spinal epidural" OR CSE) AND (vasopressor OR vasopressors OR norepinephrine OR noradrenaline OR phenylephrine OR ephedrine) AND ("treatment" OR "therapeutic use" OR "management") AND ("high risk" OR "pre-eclampsia" OR preeclampsia OR "fetal compromise" OR "placental insufficiency" OR "placental dysfunction" OR "preterm delivery" OR "emergency cesarean"))	1182	11
Cochrane Library	("cesarean section" OR "caesarean section" OR "cesarean delivery" OR "C-section") AND ("spinal anesthesia" OR "neuraxial anesthesia" OR "combined spinal epidural" OR CSE) AND ("postspinal hypotension" OR "spinal hypotension" OR hypotension) AND (vasopressor* OR norepinephrine OR noradrenaline OR phenylephrine OR ephedrine) AND (treatment OR "therapeutic use" OR management OR intervention) AND ("high risk" OR "pre-eclampsia" OR preeclampsia OR "placental insufficiency" OR "placental dysfunction" OR "fetal compromise" OR "preterm birth" OR "emergency cesarean")	84	2
Embase	('cesarean section' OR 'cesarean delivery' OR 'caesarean section' OR 'C-section') AND ('spinal anesthesia' OR 'neuraxial anesthesia' OR 'combined spinal epidural' OR CSE) AND ('postspinal hypotension' OR 'spinal hypotension' OR hypotension) AND (vasopressor* OR norepinephrine OR noradrenaline OR phenylephrine OR ephedrine) AND (treatment OR 'therapeutic use' OR management OR intervention) AND ('high risk' OR preeclampsia OR 'pre-eclampsia' OR 'placental insufficiency' OR 'placental dysfunction' OR 'fetal compromise' OR 'preterm birth' OR 'emergency cesarean')	127	1
Total Hits		1449	19
